# Supplementary material for: Exploration of precision coregulator TR-FRET identifies diverse signatures for LXR ligands relevant to discovery of nonlipogenic ABCA1 inducers
Source: eLife. 2026 Jun 22;14:RP109146. doi: 10.7554/eLife.109146 (PMC13286573; doi:10.7554/eLife.109146)
Supplement: Figure 2—figure supplement 1—source data 1. [file elife-109146-fig2-figsupp1-data1.pdf]

Figure 2-figure supplement 1-souce data 1. Summary of NR Panel Parameters		
**Lanthascreen**		
**Receptor**	**Coactivator/Acceptor**	**Donor**
FXR	SRC2-2-fluorescein	α-GST-Terbium
PXR	SRC1-4-fluorescein	α-GST-Terbium
PPARδ	C33-fluorescein	α-GST-Terbium
RXRα/β	D22-fluorescein	α-GST-Terbium
RARα	D22-fluorescein	α-GST-Terbium
RARβ	SRC2-2-fluorescein	α-GST-Terbium
