# Supplementary material for: Exploration of precision coregulator TR-FRET identifies diverse signatures for LXR ligands relevant to discovery of nonlipogenic ABCA1 inducers
Source: eLife. 2026 Jun 22;14:RP109146. doi: 10.7554/eLife.109146 (PMC13286573; doi:10.7554/eLife.109146)
Supplement: Figure 2—figure supplement 1—source data 2. [file elife-109146-fig2-figsupp1-data2.pdf]

Figure 2-figure supplement 1-source data 2. Coregulator Peptide Sequences				
**Vendor**	Catalog No.	Coregulator	Sequence	MW (Da.)
Anaspec	AS-62152	SRC1-btn	Biotin-CPSSHSSLTERHKILHRLLQEGSPS	3026.6
Thermo Fischer	PV4386	FL-D22	Fluorescein-LPYEGSLLLKLLRAPVEEV	2499
Thermo Fischer	PV4549	FL-TRAP220/DRIP-1	Fluorescein-NTKNHPMLMNLLKDNPAQD	2554
Thermo Fischer	PV4582	FL-SRC1-4	Fluorescein-GPQTPQAQQKSLLQQLLTE	2465.9
Thermo Fischer	PV4586	FL-SRC2-2	Fluorescein-LKEKHKILHRLLQDSSSPV	2585
Thermo Fischer	PV4421	FL-PGC1a	Fluorescein-EAEEPSLLKKLLLAPANTQ	2424
Thermo Fischer	PV4606	FL-C33	Fluorescein-HVEMHPLLMGLLMESQWGA	2539
Thermo Fischer	PV4624	FL-NCoR ID2	Fluorescein-DPASNLGLEDIIRKALMGSFDDK	2865
Thermo Fischer	PV4423	FL-SMRT ID2	Fluorescein - HASTNMGLEAIIRKALMGKYDQW	2993
