# Supplementary material for: Exploration of precision coregulator TR-FRET identifies diverse signatures for LXR ligands relevant to discovery of nonlipogenic ABCA1 inducers
Source: eLife. 2026 Jun 22;14:RP109146. doi: 10.7554/eLife.109146 (PMC13286573; doi:10.7554/eLife.109146)
Supplement: Figure 4—source data 1. [file elife-109146-fig4-data1.docx]

Figure 4-source data 1. Data used in correlation plots and heatmaps.

Potency (-log EC_50_)
