# Supplementary material for: Exploration of precision coregulator TR-FRET identifies diverse signatures for LXR ligands relevant to discovery of nonlipogenic ABCA1 inducers
Source: eLife. 2026 Jun 22;14:RP109146. doi: 10.7554/eLife.109146 (PMC13286573; doi:10.7554/eLife.109146)
Supplement: Figure 9—source data 1. [file elife-109146-fig9-data1.docx]

|  | SRC1α | NCOR2α | SMRT2α | SRC1β | NCOR2β | SMRT2β | HepG2-SRE | ABCA1 |
| --- | --- | --- | --- | --- | --- | --- | --- | --- |
| T0901317 | 93.37 | 91.70310528 | 83.05925029 | 97.84 | 92.3401757 | 84.98235327 | 91.33 | 99.73395993 |
| GW3965 | 37.86 | 99.05217728 | 92.36071088 | 95.64 | 92.2684161 | 92.51996642 | 71.74 | 81.39386888 |
| LXR-623 | 34.21107858 | 91.73073033 | 100 | 92.41087332 | 103.7005432 | 100.3928571 | 48.5261 | 83.67238173 |
| MK9 | 8.83084746 | 95.10137458 | 87.90804035 | 93.87254175 | 102.6843752 | 87.86368508 | 61.4664 | 83.9208158 |
| DMHCA | 59.95292422 | 100 | 99.37736369 | 75.40275393 | 106.3910195 | 98.67667443 | 18.8 | 85.48171348 |
| CL2-57 | 19.18666619 | 109.63873 | 99.98347371 | 27.25145715 | 107.4349509 | 99.35669185 | 34.43488083 | 80.44624312 |
| CL3-3 | 60.31092311 | 100 | 87.87649944 | 85.30087579 | 104.3122482 | 88.55637075 | 78.70930252 | 123.7078516 |
| RGX-104 | 71.25484176 | 99.78958921 | 93.44698343 | 100.1584679 | 103.279539 | 81.95589688 | 87.38555279 | 82.47792462 |
| BE1218 | 38.85784994 | 100 | 91.79031714 | 98.36273939 | 104.8578543 | 81.55595221 | 66.3 | 62.85320742 |
| XL652 | 59.04699685 | 100 | 97 | 100.793569 | 107.2985743 | 102.3253449 | 25 | 51.53916966 |
| ALX101 | 38.79156306 | 100 | 87.29628585 | 85.27073661 | 105.3830721 | 91.13807062 | 72.0265088 | 88.84231882 |
| WA | 70 | 100 | 100 | 70 | 100 | 100 | 70 | 70 |
| AZ876 | 101.3172196 | 79.44936072 | 46.06574835 | 98.95475658 | 78.35684164 | 63.48303759 | 87.95632601 | 106.7013798 |
| GSK3987 | 82.51218782 | 97.67143034 | 69.85630792 | 95.28317524 | 97.40247735 | 84.02254692 | 127.4639312 | 101.9248339 |
| FA | 101 | 64 | 46 | 100 | 22 | 62 | 127 | 123 |
| XL041 | 82.80769567 | 141.6 | 152.5 | 95.37246141 | 128.8722945 | 152.5495087 | 22.5 | 44.08501745 |
| PA | 101 | 142 | 152 | 100 | 128 | 152 | 50 | 50 |
| SR9238 | 0 | 64.52046255 | 64.47431302 | 0 | 21.90467677 | 69.21964091 | -22.158 | -34.0745463814932 |
| IA | 0 | 64 | 46 | 0 | 22 | 62 | 0 | 0 |
| GSK2033 | 0 | 97.67143034 | 92.90227612 | 0 | 57.69163298 | 93.36052342 | 5 | -33.2927421891987 |
| HA | 0 | 100 | 100 | 0 | 100 | 100 | 0 | 0 |
| SA | 0 | 142 | 152 | 0 | 128 | 152 | -22 | -34 |
| NLAI | 8.8 | 140 | 152 | 100 | 78 | 63 | 30 | 123 |
