# Supplementary material for: Exploration of precision coregulator TR-FRET identifies diverse signatures for LXR ligands relevant to discovery of nonlipogenic ABCA1 inducers
Source: eLife. 2026 Jun 22;14:RP109146. doi: 10.7554/eLife.109146 (PMC13286573; doi:10.7554/eLife.109146)
Supplement: Peer review history

# Editor report: eLife Assessment

**DOI:** 10.7554/eLife.109146.3.sa0

**Authors:** Warren Andrew Andayi

**Affiliations:** Murang'a University of Technology Kenya

**Keywords:** Compelling, Important

The study curated a set of Liver X receptor ligands that may guide the design of future drugs that activate the Liver X receptor as potential therapeutics for cardiovascular disease, Alzheimer's and type 2 diabetes, without inducing mechanisms that promote fat/lipid production. The authors also present improved multiplexed precision CRT (coregulator TR-FRET) and cellular assays which allows measurement of ligand potencies to displace corepressors in the presence of coactivators, which cannot be achieved in a regular CRT assay. This makes the evidence presented **compelling** as it stretches beyond the current state-of-the-art, and these **important** findings are expected to have practical implications in many sub-fields and remain of interest to scientists working in cell and molecular biology, drug discovery, medicinal chemistry and pharmacology.

# Referee report: Reviewer #1 (Public review)

**DOI:** 10.7554/eLife.109146.3.sa1

Summary:

This important study functionally profiled ligands targeting the LXR nuclear receptors using biochemical assays in order to classify ligands according to pharmacological functions. Overall, the evidence is solid, but nuances in the reconstituted biochemical assays and cellular studies and terminology of ligand pharmacology limit the potential impact of the study. This work will be of interest to scientists interested in nuclear receptor pharmacology.

Strengths:

(1) The authors rigorously tested their ligand set in CRTs for several nuclear receptors that could display ligand-dependent cross-talk with LXR cellular signaling and found that all compounds display LXR selectivity when used at ~1 µM.

(2) The authors tested the ligand set for selectivity against two LXR isoforms (alpha and beta). Most compounds were found to be LXRbeta-specific.

(3) The authors performed extensive LXR CRTs, performed correlation analysis to cellular transcription and gene expression, and classification profiling using heatmap analysis-seeking to use relatively easy-to-collect biochemical assays with purified ligand-binding domain (LBD) protein to explain the complex activity of full-length LXR-mediated transcription.

Comments on revisions:

The authors have addressed the comments from the prior round of review with care. I find the revised manuscript significantly strengthened.

# Referee report: Reviewer #2 (Public review)

**DOI:** 10.7554/eLife.109146.3.sa2

Summary:

In this manuscript by Laham and co-workers, the authors profiled structurally diverse LXR ligands via a coregulator TR-FRET (CRT) assay for their ability to recruit coactivators and kick off corepressors, while identifying coregulator preference and LXR isoform selectivity.

The relative ligand potencies measured via CRT for the two LXR isoforms were correlated with ABCA1 induction or lipogenic activation of SRE depending on cellular contexts (i.e, astrocytoma or hepatocarcinoma cells). While these correlations are interesting, there is some leg room to improve the quantitative presentation of these correlations. Finally, the CRT signatures were correlated with the structural stabilization of the LXR: coregulator complexes. In aggregate, this study curated a set of LXR ligands with disparate agonism signatures that may guide the design of future nonlipogenic LXR agonists with potential therapeutic applications for cardiovascular disease, Alzheimer's and type 2 diabetes, without inducing mechanisms that promote fat/lipid production.

Strengths:

This study has many strengths, from curating an excellent LXR compound set, to the thoughtful design of the CRT and cellular assays. The design of a multiplexed precision CRT (pCRT) assay that detects corepressor displacement as a function of ligand-induced coactivator recruitment is quite impressive as it allows measurement of ligand potencies to displace corepressors in the presence of coactivators, which cannot be achieved in a regular CRT assay that looks at coactivator recruitment and corepressor dissociation in separate experiments.

Comments on revisions:

These weaknesses have been satisfactorily addressed by the authors in the revised preprint.

# Author comment: Author response

**DOI:** 10.7554/eLife.109146.3.sa3

**Authors:** Megan S Laham, Martha S Ackerman-Berrier, Fahmida Alam, Sarah Turner, Ganga Reddy Velma, Christopher Penton, Soumya Reddy Musku, Manan Rana, Senthil Kumar, Anandhan Annadurai, Maha Ibrahim Sulaiman, Nina Ma, Gregory RJ Thatcher

**Affiliations:** University of Arizona Tucson United States; University of Arizona Tucson United States; University of Arizona Tucson United States; University of Arizona Tucson United States; University of Arizona Tucson United States; University of Arizona Tucson United States; University of Arizona Tucson United States; University of Arizona Tucson United States; University of Arizona Tucson United States; University of Arizona Tucson United States; University of Arizona Tucson United States; University of Arizona Tucson United States; University of Arizona Tucson United States

The following is the authors’ response to the original reviews.

**Public Reviews:**

**Reviewer #1 (Public review):**

Summary:

This important study functionally profiled ligands targeting the LXR nuclear receptors using biochemical assays in order to classify ligands according to pharmacological functions. Overall, the evidence is solid, but nuances in the reconstituted biochemical assays and cellular studies and terminology of ligand pharmacology limit the potential impact of the study. This work will be of interest to scientists interested in nuclear receptor pharmacology.

Strengths:

(1) The authors rigorously tested their ligand set in CRTs for several nuclear receptors that could display ligand-dependent cross-talk with LXR cellular signaling and found that all compounds display LXR selectivity when used at ~1 µM.

(2) The authors tested the ligand set for selectivity against two LXR isoforms (alpha and beta). Most compounds were found to be LXRbeta-specific.

The majority of ligands were found to be LXRβ-selective; however, examples of non-selective and LXRα-selective ligands were identified. It should be noted that this is a small compound set of literature ligands with reasonable structural diversity.

(3) The authors performed extensive LXR CRTs, performed correlation analysis to cellular transcription and gene expression, and classification profiling using heatmap analysis-seeking to use relatively easy-to-collect biochemical assays with purified ligand-binding domain (LBD) protein to explain the complex activity of full-length LXR-mediated transcription.

Weaknesses:

(1) The descriptions of some observations lack detail, which limits understanding of some key concepts.

Changes to the submitted manuscript hopefully add clarity. Several observations reinforce aspects of the literature and are a corollary of the observation that the majority of ligands with agonist activity more strongly stabilize/induce coactivator-bound complexes with LXRβ. This results in general LXRβ selectivity for agonists and also more variability in the response of LXRα to different ligand chemotypes. The most significant observations were for partial agonists that stabilize corepressor binding, in particular of the complex with LXRα.

(2) The presence of endogenous NR ligands within cells may confound the correlation of ligand activity of cellular assays to biochemical assay data.

This is generally a confounding factor for ligands with apparent antagonist activity and is a source of ambiguity in designating inverse agonists across the nuclear receptor research field. Theoretically, this could also impact weak and partial agonists; however, this requires further study.

(3) The normalization of biochemical assay data could confound the classification of graded activity ligands.

Normalization to TO (100%) and vehicle (0%) is applied to most data. It is not clear how this confounds data interpretation. TO is a very reliable and reproducible agonist without significant bias towards LXR isoforms.

(4) The presence of >1 coregulator peptide in the biplex (n=2 peptides) CRT (pCRT) format will bias the LBD conformation towards the peptide-bound form with the highest binding affinity, which will impact potency and interpretation of TR-FRET data.

Multiplex assays must be optimized to balance binding affinity of the coregulator peptides (bear in mind these are somewhat-artificial small peptide constructs that are hoped to reflect binding of the much larger coregulator protein itself). Since the dominant theory of NR tissue-selectivity is based on the cellular availability (read concentration) of coregulators, this balance exists in a cellular context.

(5) Correlation graphical plots lack sufficient statistical testing.

Correlations are now supported by statistical data and we have added hierarchical clustering analysis.

(6) Some of the proposed ligand pharmacology nomenclature is not clear and deviates from classifications used currently in the field (e.g., hard and soft antagonist; weak vs. partial agonist, definition of an inverse agonist that is not the opposite function to an agonist).

Classifications used currently in the field vary from one NR to another and the use of partial and inverse agonist, in particular, is usually qualitative, unclear, and often misleading. We expand on these classifications with respect to our use of labels to classify pCRT response to LXR ligands. In agreement with the reviewer, we have replaced IA (inverse agonist) with (RA) reverse agonist as a label specifically associated with pCRT analysis.

**Reviewer #2 (Public review):**

Summary:

In this manuscript by Laham and co-workers, the authors profiled structurally diverse LXR ligands via a coregulator TR-FRET (CRT) assay for their ability to recruit coactivators and kick off corepressors, while identifying coregulator preference and LXR isoform selectivity.

The relative ligand potencies measured via CRT for the two LXR isoforms were correlated with ABCA1 induction or lipogenic activation of SRE, depending on cellular contexts (i.e, astrocytoma or hepatocarcinoma cells). While these correlations are interesting, there is some leeway to improve the quantitative presentation of these correlations. Finally, the CRT signatures were correlated with the structural stabilization of the LXR: coregulator complexes. In aggregate, this study curated a set of LXR ligands with disparate agonism signatures that may guide the design of future nonlipogenic LXR agonists with potential therapeutic applications for cardiovascular disease, Alzheimer's, and type 2 diabetes, without inducing mechanisms that promote fat/lipid production.

Strengths:

This study has many strengths, from curating an excellent LXR compound set to the thoughtful design of the CRT and cellular assays. The design of a multiplexed precision CRT (pCRT) assay that detects corepressor displacement as a function of ligand-induced coactivator recruitment is quite impressive, as it allows measurement of ligand potencies to displace corepressors in the presence of coactivators, which cannot be achieved in a regular CRT assay that looks at coactivator recruitment and corepressor dissociation in separate experiments.

Weaknesses:

I did not identify any major weaknesses.

**Recommendations for the authors:**

**Reviewer #1 (Recommendations for the authors):**

(1) Page 2. "The endogenous ligands ... activate LXR via canonical or alternate mechanisms." What is an alternate mechanism?

Small modifications to Fig. 1 caption identify a mechanism alternative to the canonical mechanism: LXR transcriptional complexes are RXR heterodimers that can be activated by a canonical mechanism of coregulator recruitment or an alternative de-repression mechanism

(2) Page 5: "Notably, the 25 amino acid SRC-1 peptide is the only coactivator tested for LXR binding that has the fluorophore remote from the coactivator peptide." What does this mean, and could it influence the results?

The sentence has been expanded to clarify the meaning. Notably, the 25 amino acid SRC-1 peptide is the only coactivator, amongst those tested for LXR binding, which has the fluorophore remote from the coactivator peptide: i.e., the only coactivator tested that uses a fluorophore labeled anti-tag antibody to bind the tagged coactivator rather than a fluorophore-labeled coactivator. In methods based on fluorescent tags (CRT, TR-FRET, fluorescence polarization, etc.), a fluorophore that interacts directly with the receptor can generate a maximal signal that differs depending on this interaction: i.e. the identity of the coregulator used in CRT can influence the response. As seen in Figures 6 and S6, maximal response is dependent on ligand and coregulator.

(3) Page 5: "The [CRT] assay measures the EC50 for coactivator recruitment, a measure of ligand binding affinity." The dose-dependent activity in the CRT assays is more classically defined as a functional "potency", not "affinity".

The text is changed to remove “measure of affinity”: The assay measures the ligand-dependent EC_50_ for ligand-induced coactivator recruitment to LXR; the affinity of the ligand for the LXR:coregulator complex contributes to this potency

(4) Page 5: "Perhaps surprisingly, considering the description of multiple LXR ligands as partial agonists, most agonists studied gave maximal response at the same level as T0, behaving as full agonists." Can the authors speculate as to why partial agonist activity is not observed in their CRT assays when it has been observed in CRT assays for other nuclear receptors?

This section has been reworded and please note the apparent partial agonist activity observed in CRT assays for multiple coactivators as shown in Figures 6 and S6 (also see (2) above). Although many LXR ligands have been reported to display partial agonist activity, most agonists studied in this specific biotin-SRC-1 CRT assay, gave maximal response at the same level as T0, behaving as full agonists.

(5) Page 5: "Conformational cooperativity of LBD residues beyond these two amino acids leads to different conformations of Leu274 and Ala275 that generally favor ligand binding to LXRβ." Where are these residues located? Why are they important?

We have simplified this paragraph that introduces the interesting observations and interpretation of Ding et al. to illustrate potential contributions to isoform selectivity: The ligand binding pockets of the two LXR isoforms differ by only one amino acid located in helix-3. (H3: LXRα-Val263 and LXRβ-Ile277) Interestingly, correction of this difference by mutation of these residues to alanine (V263A and I277A) was observed to lower, but not to ablate isoform selectivity in reporter assays.[108] Supported by modeling studies, this observation by Ding et al. led to the suggestion that conformational cooperativity of LBD residues beyond these two amino acids, generally favors ligand binding to LXRβ. Therefore, most reported ligands, including those examined in the current work, are LXRβ-selective or non-selective.

(6) Some correlation plots are described to show "poor" correlations without showing the underlying statistical fits. All correlation plots should show Pearson and Spearman correlation coefficients and p-values within the figures.

This section of the manuscript has been completely reworked with full correlation analysis and stats . There is no substantive change in data interpretation.

(7) The normalization of TR-FRET data could introduce undesired bias when comparing activities. The methods section should provide more details about normalization of CRT data, including stating whether the control compounds' activity data were collected on the same CRT 384-well plate on the same day, or different plates, or different days, etc.

This is now clarified in SI materials and methods section. In-plate controls are always used.

(8) The authors describe their pCRT assay as "multiplex", whereas "biplex" might be more accurate, as they only used two peptides.

Biplex is commonly used referring to qPCR. Bio-Plex is a commercial version of an antibody assay. Duplex is obviously a term used in nucleic acid research. Therefore, multiplex is a simpler, more generic term that we feel is suitable and can be extended to add a third coregulator.

(9) The pCRT assays use the same peptide concentrations (200 nM). However, the peptides will have different affinities for the LBD, which may bias ligand-dependent pCRT profiles. The peptide that binds with higher affinity in the absence of ligand will bias the LBD conformation and impact ligand affinity. Can the authors comment on any limitations of the pCRT approach vs. a normal CRT? Did the authors perform any optimization to see if increasing peptide concentrations (>200 nM) or having different concentrations (e.g., 400 nM SRC1 and 200 nM NCorR2) influences the pCRT data, extracted parameters, correlations, etc.?

As we write in the Limitations section, our assays are focused on ligand-dependence, whereas other excellent studies focus more on coregulator-dependence. The length and affinity of peptide constructs varies and therefore it is important to “balance” corepressor and coactivator concentrations. The most important conclusions from our pCRT assays concern the ability of some ligands to stabilize corepressor binding in the monoplex CRT and the universal ability of coactivator complex stabilization to eject the corepressor in the multiplex assay. Furthermore, without measurements and correlations in “natural” cellular contexts, the CRT data obtained in cell-free conditions is somewhat artificial. We evaluated a range of peptide concentrations to assess signal-to-background and overall assay performance. Each new receptor added to the panel underwent rigorous optimization to establish robust and reliable assay conditions. This included identifying a suitable positive control for each receptor, determining the optimal coregulator selection and concentration, and refining other key parameters such as buffer composition and total well volume. The concentrations reported represent the optimized balance—producing a strong, reproducible signal without oversaturation or disproportionate contribution from any individual assay component.

(10) Page 11. The authors introduce a few ligand classification terms that are not standard in the field and unclear: "soft" vs. "hard" antagonist, "weak" vs. "partial" agonist, and their definition of an inverse agonist that, in classical pharmacologic terms, should have an opposite (inverse) function to an agonist. Furthermore, the presence of endogenous LXR ligands within cells may confound the correlation of ligand activity of cellular assays to biochemical assay data. See the following paper for an example of ligand-dependent classification and activation mechanisms when there are endogenous cellular ligands at play: https://elifesciences.org/articles/47172

The paragraph discussing nomenclature went through many iterations of terminology and a further paragraph was removed that discussed problems with ligand classification in the broader field of NR pharmacology: this has now been added back. We apologise for not citing the excellent Strutzenberg et al. paper on RORa pharmacology, which is now included. In this paper, Griffin and co-workers also use terms that are not standard in the field, such as “silent agonist”, which covers, in part, ligands that we describe as “weak agonists”. A standard, definitive lexicon of terms across NRs is unfortunately problematic. We have added 2 paragraphs:

The nomenclature for NR ligands often lacks precision and differs across NR classes. SERM (a subset of selective NR modulator) is used to describe varied families of ER ligands that show tissue-selective agonist and/or antagonist actions. Unfortunately, “partial agonist” is also widely used to describe SERMs, even though its use is usually pharmacologically incorrect and biased agonist may be a more accurate label.[124] The majority of reported ER ligands are SERMs, even some that cause ER degradation, because they are transcriptionally active. Consequently, the term “pure antagonist” (PA) has been used to differentiate transcriptionally null ligands[125]; although, pure antagonist/antiestrogen was originally introduced to describe antagonism of both AF1 and AF2 functions.[90]

Elegant work by Griffin’s team on RAR-related orphan receptor C (RORɣ) is interesting, because it used a combination of HDX-MS and CRT and defined categories of RORɣ ligands.[126] In addition to full agonist, “silent agonist” was introduced to include endogenous and synthetic partial agonists; although, by definition, partial agonists should antagonize full agonists. On the antagonist side of the spectrum, “active antagonist” was used to describe ligands that reduce cellular activity to baseline; and “inverse agonist” for ligands that reduce cellular transcription below baseline and induce recruitment of corepressors. Curiously, inverse agonist has almost never been used to describe ER ligands and is used frequently for other NR ligands, mostly for ligands that reduce transcription below baseline, without any evidence for corepressor recruitment. GSK2033 and SR9238 show inverse agonist activity in cells (Figs 3, 5); however, neither is capable of recruiting SMRT2 or NCOR2 to LXR (Fig. 7).

(11) Figure 9A and Figure S8. Could hierarchical clustering analysis be used to more rigorously compare the activities of the ligands?

We have now added hierarchical clustering analysis (Figs 4 S4). It should be noted that the value of such an analysis is much higher when the number of ligands is increased.

(12) How does cellular potency correlate to pCRT vs. CRT potencies? Does pCRT better explain cellular potency?

We have added this specific correlation (multiplex CRT vs. monoplex CRT).

(13) The authors should provide an SI table of parameters (potency values) used for correlation and heatmap analyses.

Tables have been added to SI accordingly.

**Reviewer #2 (Recommendations for the authors):**

This manuscript has many strengths, but can still be improved by addressing the following critiques:

(1) I am surprised the team did not find a ligand with a higher efficacy than T0. Please would you explain why T0 seems to have maxed out ligand efficacy for both LXRalpha and LXRbeta?

Several ligands gave superior efficacy to T0 in cell-based reporter assays and in CRT assays shown in Figures 6 and S6: AZ876, BE1218, and MK9 gave maximal response higher than that of T0.

(2) In the subsection, "Activity and isoform selectivity of LXR ligands", you mentioned that "The assay measures the EC50 for coactivator recruitment, a measure of ligand binding affinity." This is incorrect. EC50 is a measure of ligand potency, not affinity.

See Reviewer-1 (3)

(3) In Figure 3 it is unclear what was used to normalize the antagonist responses in Panel F. Also, I recommend changing the y-axis of Panel F to -100 to 50 to get a better view of the response.

This has been clarified: zero is vehicle control. Change to y-axis is made.

(4) In Figure 4, the correlation R-squared values should be presented as a Table to have a better qualitative assessment of the correlations. It is challenging to judge which correlations are better by relying only on visual inspection. I also recommend moving the two panels from Figure S3 to Figure 4 as panels E and F.

Extensive changes to Figure 4 have been made in response to this comment and that of Reviewer 1, who wanted these values in the figures: Reviewer-1 points (6) and (12).

(5) In Figure 5, the fold changes in panels G, H, and I could better be presented as a bar graph. Also, the cytotoxicity of ligands needs to be assessed. For instance, in BE1218, there is a sharp decrease in fold change going from ~1 uM to ~10 uM. This will also confirm if the downward trends for SR9238 and GSK2033 are "real" and not as a result of cells dying off at higher ligand concentrations.

Across our many studies on potent NR ligands, at concentrations above 3 uM, cell growth inhibition is observed. This is true for ER ligands, such as tamoxifen, with explanations in the literature including membrane disruption and low-affinity cytoplasmic binding proteins. We include cell viability measurements in Supplemental as a specific response to the reviewer’s query. There is no loss of cell viability in HepG2 cells.

(6) Several ligands induce recruitment of coactivators but with minimal ability to displace corepressors. Physiologically, what would be the expected effect of these ligands on LXR activity?\

We have defined such ligands from pCRT analysis as weak agonists (WA); however, pCRT shows WA ligands induce corepressor loss in the presence of coactivator. Depending on coregulator balance and isoform expression and the importance of the derepression mechanism in a specific cell context, WA ligands might be expected to be differentiated from SA (strong agonist) ligands.

(7) In the subsection, "synchronous coregulator recruitment by multiplex, precision CRT" you mentioned that "For LXRbeta, the correlation between SRC1 recruitment in monoplex and multiplexed CRT is good," but the data is not shown. I think it would be better to show this data for transparency.

See query (4) and Reviewer-1. Done.

(8) In Figure 9, Panel A, the heat map is quantitated as 0-150. Is this fold change? If so, add this label to the figure legend.

It is Normalized Response as %, which is now added.

(9) In Figure 9, Panel B, please explain why in all cases, CoA-bound LXR resides at a higher energy level than the CoR-bound, and the apo LXR is at a lower energy level than the CoA-bound protein. A coregulator-bound (holo) protein structure is generally a lower energy (more stable) structure than the unbound (apo) protein. The binding of a coregulator stabilizes the protein's conformation and shifts the equilibrium towards a more thermodynamically favorable state. Using the same argument, it does not make sense to me that the CoR-bound LXR is on the same energy level as the apo LXR.

This schema reflects our observations in pCRT. No signal was observed for coactivator-bound (holo) protein in the absence of ligand; whereas, a signal was observed for corepressor-bound (holo) protein in the absence of ligand. Therefore, the CoA-bound LXR is higher energy than apo-LXR (+ unbound CoA). Conversely, the signal for CoR-bound LXR can be reduced or increased by ligands, requiring the CoA-bound LXR to be of similar energy to apo-LXR (+ unbound CoR).

(10) In the Figure 9b caption, "measured at 1uM" pertains to the concentration of ligand or coregulator? This is unclear. You should report the concentration of both ligand and coregulator.

Clarified in caption.

(11) In Figure S4, signal for SR9238 shoot up to ~300 units for ligand concentrations >3 uM. Please explain what could have contributed to this anomalous activation and why this was moved to the Supplementary File and not shown in the main figure (Figure 5).

The HepG2-SRE assay is a nano-luc reporter assay, unlike the CCF-ABCA1 that is a firefly luciferase assay. There is substantial anecdotal evidence that furimazine/nano-luc is susceptible to stabilization enhancement. The RT-PCR data presented in Fig. 5 confirms that this is an artifact for some biphenyl sulfones.
